# Successful treatment of Henoch-Schönlein purpura-associated hematochezia in a child with hemophilia A: a case report

**DOI:** 10.1186/s12887-023-03874-w

**Published:** 2023-03-02

**Authors:** Kai Feng, Chang Liu, Keqing Zhang, Jing Hao

**Affiliations:** Department of Traditional Chinese Medicine, Beijing Children’s Hospital, Capital Medicine University, National Center for Children’s Health, No.56 Nanlishi Road, Beijing, 100045 China

**Keywords:** Hemophilia A, Henoch-Schönlein purpura, Hematochezia, Gastrointestinal bleeding, Factor VIII, Case report

## Abstract

**Background:**

Henoch-Schönlein purpura (HSP) is a common form of immunological vasculitis in children. Hemophilia A is a genetic disorder and characterized by spontaneous hemorrhage or prolonged bleeding due to factor VIII deficiency. Both diseases increase the risk of bleeding, but they have different mechanisms. How should we treat patients with both diseases?

**Case presentation:**

An 8-year-old male with hemophilia A was diagnosed with HSP while receiving coagulation factor VIII replacement therapy in our hospital. Hematochezia occurred 6 days after the diagnosis of HSP. And he treated with coagulation FVIII, methylprednisolone and hemostatic drugs.

**Conclusions:**

There is no causal relationship between hemophilia A and HSP, but both diseases can cause bleeding. This child's hematochezia was caused by HSP, but hemophilia could not be ignored during the treatment. Our case report adds to the present body of knowledge about the treatment of HSP associated hematochezia in a child with hemophilia A.

## Background

Hemophilia is a kind of hereditary hemorrhagic disease with X-linked recessive inheritance caused by coagulation factor deficiency, including hemophilia A and hemophilia B. Hemophilia A with a functional defect of factor VIII (FVIII) caused by mutations of the F8 gene in locus Xq28 accounts for 80%-85% of hemophilia cases [1]. The global incidence of hemophilia A is 1 in 5000 males, and there are approximately 100 000 patients in China [2, 3]. According to the concentration of coagulation FVIII, hemophilia A is divided into severe (FVIII activity < 1%), moderate (1% < FVIII activity < 5%) and mild (5% < FVIII activity < 40%) cases. The clinical features are spontaneous bleeding or intractable bleeding after minor trauma in various parts of the body. The state-of-the-art treatment for hemophilia A is replacement therapy with human plasma-derived FVIII or recombinant coagulation FVIII.

Henoch-Schönlein purpura (HSP) is the most common form of systemic vasculitis in children, affecting 14 of every 100 000 children [4]. The early phase clinical manifestations of HSP, including purpura, arthralgia, and abdominal pain, are common. The incidence of digestive system involvement in HSP patients is 50-80% [5], which is characterized by nausea, vomiting, diarrhea, abdominal pain and gastrointestinal bleeding. Detection of gastrointestinal involvement occurs mainly as blood in stools in the early phase of HSP. Furthermore, renal disease, the onset of which may be delayed for weeks or months after the appearance of other early phase symptoms, is significantly related to gastrointestinal symptoms [6, 7].

### Case presentation

The patient was an 8-year-old male who was diagnosed as severe hemophilia A (FVIII activity < 1%) when he was 2 years old and had received coagulation FVIII replacement therapy for 6 years in our hospital. At 6 days before admission, the boy had red rash on both lower limbs and feet without abdominal pain and joint pain. He was diagnosed with HSP in the local hospital and treated with cetirizine orally for 3 days. At 3 days before admission, the rash of the child did not subside and abdominal pain occurred, and received omeprazole by intravenous injection in our hospital for 2 days. When we saw the patient, his red rash on both lower limbs and feet accompany with abdominal pain without joint pain. Bloody stool occurred on the 3rd day after hospitalization for HSP. During hospitalization, the ultrasound was done and the intravenous methylprednisolone was administered to relieve inflammation-swelling caused by HSP. Recombinant coagulation FVIII was used to treat hemophilia A and aminomethylbenzoic acid, etamsylate, haemocoagulase agkistrodon and somatostatin were used to stop bleeding. According to changes in the patient’s condition, such as the presence of hematochezia, we increased the dose of intravenous methylprednisolone from 2 mg/kg/d to 4 mg/kg/d in a timely manner, and reduced 20 mg every 2–3 days when his fecal occult blood test normal. Hemostatic drugs were stopped when no red blood cells are found in the stool. Table 1 fully depicts the timeline of drugs administered to the child and their doses. Tables 2 and 3 show the laboratory tests and gastrointestinal ultrasound results during hospitalization. The patient did not complain of fever, dizziness, palpitations or fatigue, and his vital signs (heart rate, respiratory rate, pulse rate, blood pressure and oximetry) were stable under continuous monitoring system.

**Table 1:**
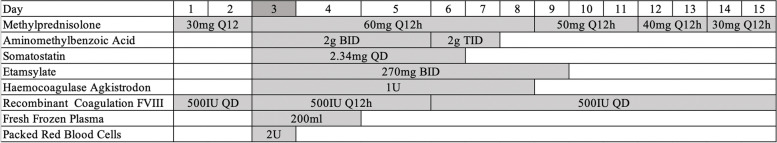
Timeline of medications. Hematochezia occurred 3rd day after the hospitalization for HSP. The boy weighs 27 kg

*Abbreviation*: *mg* Milligram, *IU *International Unit, *U *Unit of enzyme, *ml* Milliliter, *QD *Quaque die, *BID *Bis in die, *TID* Ter in die, *Q12h* once every twelve hours

**Table 2:**
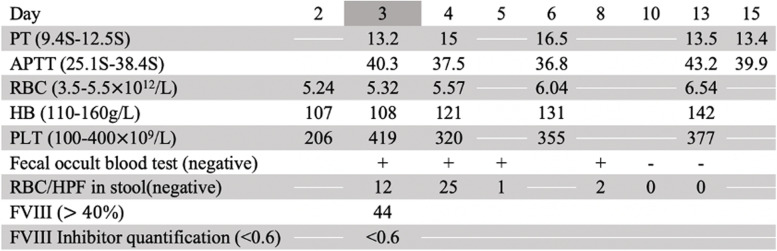
Major laboratory tests during the hospitalization

*Abbreviation*: *S* Seconds, *L *Litre. +: positive. -: negative

**Table 3:**
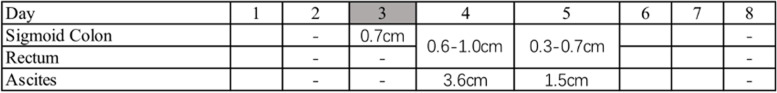
Swelling of intestinal wall by gastrointestinal ultrasound during the hospitalization

*Abbreviation*: -: negative, *cm* Centimeter

A complete blood count showed mild anemia, and fresh frozen plasma and packed red blood cells were provided simultaneously to treat anemia. Furthermore, biochemical examination showed no abnormal liver or renal function or electrolytes. We adjusted the dosage of recombinant coagulation FVIII and methylprednisolone according to the coagulation test, FVIII concentration and gastrointestinal ultrasound and added hemostatic drugs. On the 6th day after onset of hematochezia, the patient’s abdominal pain was relieved, and his stool test results and gastrointestinal ultrasound findings were within normal limits.. Finally, he demonstrated no signs of hemorrhage and was safely discharged from the hospital after palpable purpura disappeared.

## Discussion

The challenges in the hemophilic children are different from those in adults [8]. Early and long-term prophylaxis in children with hemophilia is the most effective method of forestalling bleeding and either preventing or reducing joint damage, and increasing the patients’ quality of life and life expectancy [9]. Hemophilia A is a rare inherited bleeding disorder characterized by deficiency of coagulation FVIII. Such patients, if not optimally treated, encounter recurrent bleeding episodes causing cumulative damage and, commonly, arthropathy [10]. Coagulation FVIII, as a coagulation replacement factor, has been used to treat and prevent hemophilia A for decades. It can prevent hemophilia A patients from bleeding symptoms, joint dysfunction and abnormal, massive postoperative bleeding caused by the disease. FVIII is an important coagulation factor in the endogenous coagulation pathway. As a cofactor of coagulation factor IXa, FVIII participates in the activation of coagulation factor X and forms an endogenous coagulation pathway. Gastrointestinal bleeding is one of the serious complications of hemophilia [11]. The most common cause of abdominal bleeding in hemophilia is gastrointestinal mucosal bleeding [12]. Diagnostic imaging is necessary to differentiate between various causes of abdominal bleeding. At this time, prophylactic dosing is mostly based on the Swedish regimen of 25 IU/kg thrice weekly or every other day in pediatric patients [13, 14].

HSP is a systemic vasculitis involving small blood vessels, most notably the skin, gastrointestinal tract, and glomeruli, accompanied by arthralgia or arthritis. While the exact cause of HSP remains unknown, several studies have reported infections and imbalance of immune and genetic factors as causes of HSP [15–17]. HSP can lead to severe complications, such as intussusception, gastrointestinal bleeding, or end-stage renal disease, although it is considered a self-limiting disease [18]. Treatment of HSP includes controlling the acute symptoms and influencing factors of prognosis, such as acute joint pain, abdominal pain and renal damage.

Symptomatic or occult gastrointestinal bleeding, such as rectal bleeding, melena, and hematemesis, is observed in 50% of children with HSP [19]. Glucocorticoids can quickly relieve gastrointestinal symptoms and shorten the duration of abdominal pain. [20]. Corticosteroids, given early in the course of illness, seem to produce consistent benefits for gastrointestinal bleeding, in children suffering from HSP [18]. Remarkably, our patient also had congenital malrotation of the intestine, which could increase the risk of gastrointestinal bleeding [21, 22].

Before the diagnosis of HSP, the boy received coagulation FVIII replacement therapy according to the Swedish regimen. Then, we increased the doses of coagulation FVIII and methylprednisolone and used hemostatic drugs to control bleeding when he presented with hematochezia. Ultimately, he demonstrated no signs of hemorrhage and was safely discharged from the hospital. Both diseases increase the risk of bleeding. The child receiving coagulation factor VIII replacement therapy in our hospital regularly, and the activity of FVIII and coagulation test were normal during the period. Therefore, we judge that hematochezia is mainly caused by HSP. we adjusted the dosage of recombinant coagulation FVIII and hemostatic drugs according to the coagulation test and FVIII concentration, on the other hand, intravenous methylprednisolone was administered to relieve abdominal pain caused by HSP energetically. There are others publications in the literature about HSP in a patient with hemophilia A [23, 24]. But these cases were not accompanied by gastrointestinal bleeding and there is no description of how to treat bleeding in HSP-associated hematochezia with hemophilia A.

## Conclusion

There is no causal relationship between hemophilia A and HSP, but both diseases can cause bleeding. The patient with Hemophilia A who developed abdominal pain and hematochezia with HSP was successfully treated with recombinant FVIII provided under the Swedish regimen, high dose methylprednisolone, and hemostatic drugs. Our report adds to the present body of knowledge about the treatment of HSP-associated hematochezia in a patient with hemophilia A.

### Drug information involved in the text

**Methylprednisolone** (Methylprednisolone Sodium Succinate for Injection, Pfizer Manufacturing Belgium NV, Belgium).

**Recombinant coagulation FVIII** (Recombinant Human Coagulation Factor VIII for Injection, Bayer HealthCare LLC, America).

**Aminomethylbenzoic Acid** (Aminomethylbenzoic Acid, Yangzhou Zhongbao Pharmaceutical Co., Ltd, China).

**Etamsylate** (Etamsylate, Yangzhou Zhongbao Pharmaceutical Co., Ltd, China).

**Haemocoagulase Agkistrodon** (Haemocoagulase Agkistrodon, Beijing Konruns Pharmaceutical Co., Ltd, China).

**Somatostatin** (Somatostatin for Iinjection, ALFASIGMA.S.p.A, Italy).

## Data Availability

Data sharing is not applicable to this article as no datasets were generated or analysed during the current study.

## Abbreviations

HSP: Henoch-Schönlein purpura
FVIII: Factor VIII
PT: Prothrombin time
APTT: Activated partial thromboplastin time
RBC: Red blood cell
HB: Hemoglobin
PLT: Platelet
